# Changes in Aphid Host Plant Diet Influence the Small-RNA Expression Profiles of Its Obligate Nutritional Symbiont, *Buchnera*

**DOI:** 10.1128/mBio.01733-19

**Published:** 2019-11-19

**Authors:** Margaret W. Thairu, Allison K. Hansen

**Affiliations:** a Department of Entomology, University of California, Riverside, Riverside, California, USA; b Department of Bacteriology, University of Wisconsin, Madison, Madison, Wisconsin, USA; Cornell University; University of Hawaii at Manoa

**Keywords:** sRNA, *Acyrthosiphon pisum*, *Buchnera*, regulation, pea aphid, reduced genome

## Abstract

Plants are a difficult food resource to use, and herbivorous insects have evolved a variety of mechanisms that allow them to fully exploit this poor nutritional resource. One such mechanism is the maintenance of bacterial symbionts that aid in host plant feeding and development. The majority of these intracellular symbionts have highly eroded genomes that lack many key regulatory genes; consequently, it is unclear if these symbionts can respond to changes in the insect’s diet to facilitate host plant use. There is emerging evidence that symbionts with highly eroded genomes express small RNAs (sRNAs), some of which potentially regulate gene expression. In this study, we sought to determine if the reduced genome of the nutritional symbiont (*Buchnera*) in the pea aphid responds to changes in the aphid’s host plant diet. Using transcriptome sequencing (RNA-seq), *Buchnera* sRNA expression profiles were characterized within two *Buchnera* life stages when pea aphids fed on either alfalfa or fava bean. Overall, this study demonstrates that *Buchnera* sRNA expression changes not only with life stage but also with changes in aphid host plant diet. Of the 321 sRNAs characterized in this study, 47% were previously identified and 22% showed evidence of conservation in two or more *Buchnera* taxa. Functionally, 13 differentially expressed sRNAs were predicted to target genes related to pathways involved in essential amino acid biosynthesis. Overall, results from this study reveal that host plant diet influences the expression of conserved and lineage-specific sRNAs in *Buchnera* and that these sRNAs display distinct host plant-specific expression profiles among biological replicates.

## Abstract (precis)

In general, the genomes of intracellular bacterial symbionts are reduced compared to those of free-living relatives and lack many key regulatory genes. Many of these reduced genomes belong to obligate mutualists of insects that feed on a diet that is deficient in essential nutrients, such as essential amino acids. It is unclear if these symbionts respond with their host to changes in insect diet, because of their reduced regulatory capacity. Emerging evidence suggests that these symbionts express small RNAs (sRNAs) that regulate gene expression at the posttranscriptional level. Therefore, in this study, we sought to determine if the reduced genome of the nutritional symbiont *Buchnera* in the pea aphid responds to changes in the aphid’s host plant diet. This study demonstrates for the first time that *Buchnera* sRNAs, some conserved in two or more *Buchnera* lineages, are differentially expressed when aphids feed on different plant species and potentially target genes within essential amino acid biosynthesis pathways.

## INTRODUCTION

Herbivorous insects are faced with the challenge of using a food resource that contains defensive compounds and varies in nutritional profiles both spatially and temporally (1). Consequently, insect herbivores have evolved a diversity of mechanisms that facilitate host plant use (1, 2). One mechanism that has aided various phytophagous insects in using plants as nutrient resources is the acquisition of microbial symbionts (3–6). Within the insect order Hemiptera, many examples of such bacterial symbioses are widely found (7). In the hemipteran suborders Auchenorrhyncha and Sternorrhyncha, many insects have obligate nutritional symbionts that are housed intracellularly in specialized insect cells (bacteriocytes) within the insect’s body (3, 7). These obligate intracellular symbionts have highly eroded genomes that lack many key regulatory genes (8, 9). Consequently, it is unclear if intracellular symbionts can respond to changes in insect diet to facilitate host plant use, especially in comparison to extracellular symbionts that often directly interface with the plant material and have larger, more dynamic genomes.

Within Hemiptera, the partnership between Acyrthosiphon pisum (pea aphid) and the bacterium *Buchnera* is one of the best-characterized nutritional, intracellular symbioses within insects. In this symbiosis, each partner depends on the other for the production of amino acids (10–12). The symbiont *Buchnera* possesses the majority of genes within pathways for essential amino acid biosynthesis (13), which are not possessed by the aphid (14). The aphid possesses genes that are predicted to be vital in the regulation of this microbial symbiosis. These aphid genes include those that are involved in amino acid transport, ammonia recycling, synthesis of amino donors, and metabolite intermediates, as well as the terminal steps for essential amino acid biosynthesis that are missing from the *Buchnera* genome (11, 12, 14–16). Homologs of these aphid genes are also upregulated in bacteriocytes from other hemipteran taxa, suggesting that host control of these intracellular symbioses is widespread among hemipterans (17–20). These insect genes potentially can respond to host-plant diet for example; Kim et al. (21) observed the upregulation of several of these collaborating aphid genes in bacteriocytes when aphids fed on their suboptimal, specialized host plant, alfalfa (Medicago sativa), compared to their universal host plant, fava bean (Vicia faba).

Currently, there is limited evidence that supports the role of intracellular symbionts responding to changes in insect diet to facilitate host plant use. For example, *Buchnera* displays negligible gene expression responses at the mRNA level when aphids feed on an artificial diet or plant material that has been spiked with different concentrations of amino acids (22, 23). However, Viñuelas and colleagues (24) demonstrated that *Buchnera’s* pLeu plasmid can respond to changes in the aphid’s diet by increasing plasmid copy number and upregulating the genes on the plasmid (*leuABCD*, *repA1*, *repA2*, and *yqhA*) in response to limited concentrations of leucine in an artificial diet. These results suggest that in the face of genome reduction, *Buchnera* displays limited transcriptional control on its chromosome in response to diet variation; however, other regulatory strategies may still be maintained via plasmids. Currently, it is unknown if posttranscriptional regulation is important in *Buchnera* in response to host plant diet.

Posttranscriptional strategies of gene regulation, such as small RNAs (sRNAs), have been widely observed throughout all domains of life (25–29), and emerging evidence supports the role of regulatory sRNAs in intracellular bacterial symbiont gene regulation (30). For example, sRNAs expressed from the tick symbionts Rickettsia prowazekii and Rickettsia conorii are hypothesized to be important in facilitating host niche adaptation (31, 32). In *Buchnera*, sRNAs are hypothesized to be important in regulating genes at the posttranscriptional level when *Buchnera* transitions between different life stages (33, 34). In turn, there is potential for *Buchnera* sRNAs to aid in regulating essential amino acid and vitamin biosynthesis pathways in response to changes in nutrient demand. Changes in nutrient demand may occur when aphids feed on host plants that vary in free amino acid content as well as defensive compounds such as saponins and flavonols, which may inhibit nutrient uptake by the aphid, especially on alfalfa (M. sativa) (35–40).

In this study, we sought to determine if sRNAs expressed by the intracellular symbiont *Buchnera* respond to aphid host plant diet. Specifically, we investigated if *Buchnera* sRNAs are differentially regulated when A. pisum feeds on fava bean (V. faba) compared to alfalfa (M. sativa). These host plants were chosen because they vary in amino acid profiles and host plant defenses (35–40). Moreover, the A. pisum (LSR1) sublines used in this study display higher fitness when they feed on their “universal” host plant, fava bean (FB), than on their specialized host plant alfalfa, (ALF), and several aphid genes involved in the nutritional symbioses are differentially expressed between host plant diets (21). In this study, we use transcriptome sequencing (RNA-seq) to characterize *Buchnera* sRNA expression between the aphid’s universal (FB) and specialized (ALF) host plant diets for two different *Buchnera* life stages: (i) aphid ovarioles (referred to here as embryos [EMB]), in which early in aphid development *Buchnera* is in its extracellular proliferating state, and (ii) maternal bacteriocytes (BAC), in which *Buchnera* is in an intracellular state (41). These two *Buchnera* life stages were collected separately because it was previously shown that *Buchnera* displays differential sRNA (34) and protein expression profiles (33) between these life stages.

## RESULTS

### *Buchnera* sRNA expression profiles are influenced by aphid host plant diet and *Buchnera* life stage.

Across all 12 RNA-seq samples, ALF-BAC1 to -3, ALF-EMB1 to -3, FB-BAC1 to -3, and FB-EMB1 to -3, an average of 96% of all high-quality reads mapped to either the *Buchnera* or the aphid genome (see Table S1 in the supplemental material). Each sample was sequenced to a similar read depth, with an average in ALF samples of 2.67 × 10^7^ reads and 3.12 × 10^7^ reads in FB samples mapping to the *Buchnera* genome (Table S1).

10.1128/mBio.01733-19.2 TABLE S1 Summary of RNA-seq data. FB1 to -3 and ALF1 to -3 represent the three aphid subline replicates. Download Table S1, XLSX file, 0.01 MB. Copyright © 2019 Thairu and Hansen. 2019 Thairu and Hansen This content is distributed under the terms of the Creative Commons Attribution 4.0 International license.

A total of 322 differentially expressed sRNAs were identified among all 12 samples and consisted of 253 antisense sRNAs, 17 untranslated region (UTR) sRNAs, and 52 intergenic sRNAs. Many of these sRNAs were identified previously where 51% of antisense sRNAs, 82% of UTR sRNAs, and 12% of intergenic sRNAs were found to be conserved in two or more *Buchnera* lineages (33) (Tables S2, S3, and S4).

10.1128/mBio.01733-19.3 TABLE S2 Differentially expressed antisense sRNAs. (A) Differentially expressed antisense sRNAs identified in the comparison of ALF-BAC to FB-BAC. (B) Differentially expressed antisense sRNAs identified in the comparison of ALF-EMB to FB-EMB. (C) Differentially expressed antisense sRNAs identified in the comparison of ALF-BAC to ALF-EMB. (D) Differentially expressed antisense RNAs identified in the comparison of FB-BAC to FB-EMB. Download Table S2, XLSX file, 0.1 MB. Copyright © 2019 Thairu and Hansen. 2019 Thairu and Hansen This content is distributed under the terms of the Creative Commons Attribution 4.0 International license.

10.1128/mBio.01733-19.4 TABLE S3 Differentially expressed UTR sRNAs. (A) Differentially expressed UTR sRNAs identified in the comparison of ALF-BAC to FB-BAC. (B) Differentially expressed UTR sRNAs identified in the comparison of ALF-EMB to FB-EMB. (C) Differentially expressed UTR sRNAs identified in the comparison of ALF-BAC to ALF-EMB. (D) Differentially expressed UTR sRNAs identified in the comparison of FB-BAC to FB-EMB. Download Table S3, XLSX file, 0.01 MB. Copyright © 2019 Thairu and Hansen. 2019 Thairu and Hansen This content is distributed under the terms of the Creative Commons Attribution 4.0 International license.

**FIG 1:**
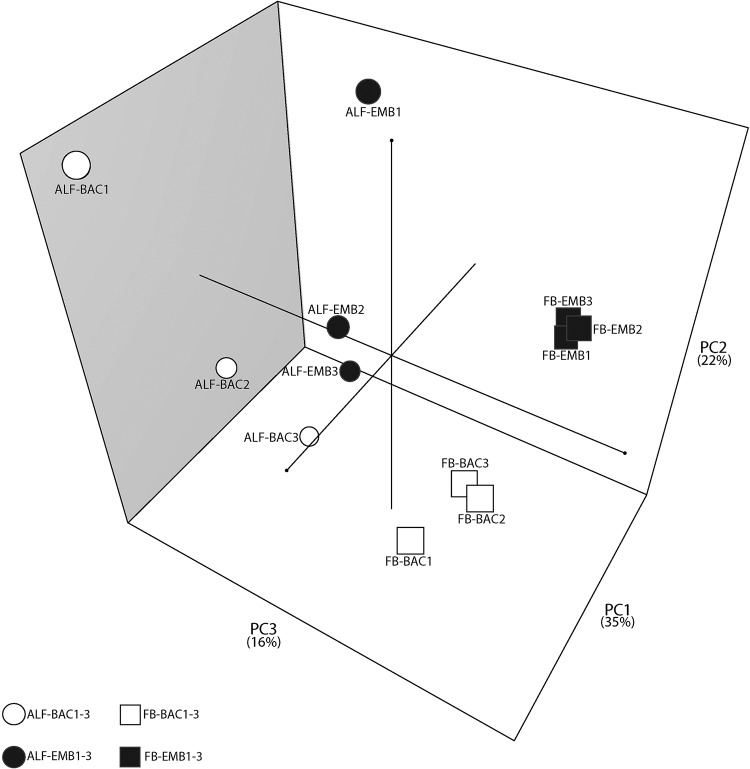
Principal-component analysis (PCA) of sRNA expression profiles for each aphid host plant treatment (fava bean [FB] and alfalfa [ALF]) and life stage group (bacteriocytes [BAC] and embryo [EMB]). Numbers after the treatment label represent the 3 biological replicates (sublines) for each aphid host plant treatment and life stage group.

After identifying expressed sRNAs using Rockhopper, principal-component analysis (PCA) and permutational multivariate analysis of variance (PERMANOVA) were run to determine how similar the *Buchnera* sRNA expression profiles were across treatments. Using PERMANOVA, the sRNA expression profiles of the groups ALF-BAC, ALF-EMB, FB-BAC, and FB-EMB were found to be significantly different from each other (*P* ≤ 0.01; *F* = 1.696). When grouped by *Buchnera* life stage, the sRNA expression profiles were found to be significantly different from each other (*P* = 0.001; *F* = 8.2119). When grouped by host plant treatment, the sRNA profiles were also found to be significantly different (*P* = 0.001; *F* = 5.0376). This segregation of sRNA expression profiles by host plant treatment and life stage was supported by the PCA ordination results (Fig. 1). The first three PCA axes explained 73% of the variation in the data set, with the first PCA axis (PC1) accounting for 35% (± 13.21 standard deviations [SD] of the variation), the second axis (PC2) accounting for 22% (± 10.43 SD), and the third axis (PC3) accounting for 16% (± 8.90 SD). Combined, these results suggest that both aphid host plant and *Buchnera* life stage have significant effects on sRNA expression profiles. Among the samples from aphids feeding on alfalfa, there was a more heterogenous response in the sRNA expression profiles (Fig. 1) than for samples from aphids feeding on fava bean. Despite this heterogenous response among ALF samples, it is important to note that there was still a significant difference in sRNA expression profiles observed between host plant treatments for both life stages. Moreover, across samples no batch effect was observed (see Text S1 [supplemental results]).

10.1128/mBio.01733-19.6 TEXT S1 Supplemental results. Download Text S1, DOCX file, 2.6 MB. Copyright © 2019 Thairu and Hansen. 2019 Thairu and Hansen This content is distributed under the terms of the Creative Commons Attribution 4.0 International license.

### Aphid host plant diet significantly affects *Buchnera* sRNA expression for genes involved in essential amino acid biosynthesis.

To determine how sRNA expression profiles (including tRNAs) differ between host plant treatments for each life stage, Rockhopper was used. Within the comparison of ALF-BAC to FB-BAC, 103 sRNAs were upregulated in the ALF-BAC treatment group and 28 sRNAs were upregulated in FB-BAC treatment group. Within the comparison of ALF-EMB to FB-EMB, 96 sRNAs were upregulated in the ALF-EMB treatment group and 74 sRNAs were upregulated in the FB-EMB treatment group (FDR-adjusted *P* value, *q* ≤ 0.05; 1.5-fold change [Table 1]).

**TABLE 1:** Number of sRNAs upregulated for each aphid host plant comparison (*q* ≤ 0.05; 1.5-fold change)

Comparison	Type of sRNA	Upregulated in ALF	Upregulated in FB
ALF-BAC vs FB-BAC	Antisense sRNA	69	17
	Intergenic sRNA	19	2
	UTR sRNA	11	0
	tRNAs		
	Sense	4	1
	Antisense	0	8
	Total	103	28
ALF-EMB vs FB-EMB	Antisense sRNA	63	56
	Intergenic sRNA	22	7
	UTR sRNA	7	2
	tRNAs		
	Sense	4	2
	Antisense	0	9
	Total	96	74

**FIG 2:**
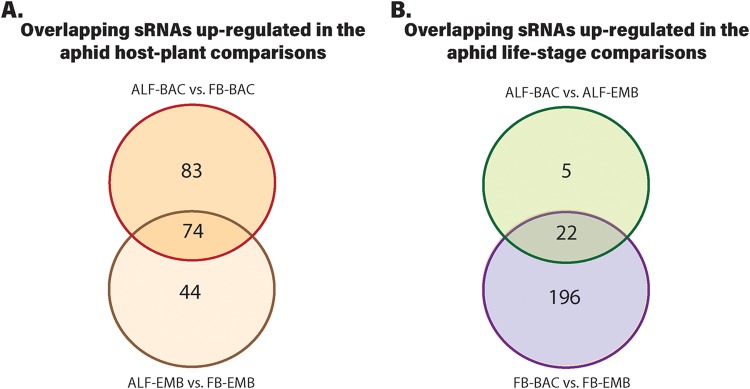
Venn diagrams showing the number of overlapping sRNAs found to be upregulated in the host plant comparisons of ALF-BAC to FB-BAC and ALF-EMB to FB-EMB (A) and the aphid life stage comparisons of ALF-BAC to AFL-EMB and FB-BAC to FB-EMB (B).

Of the 279 sRNAs (not including tRNAs) differentially expressed between the two aphid host plant treatments for both life stages (BAC: ALF-BAC versus FB-BAC) and (EMB: ALF-EMB versus FB-EMB), 74 sRNAs were found to be differentially expressed in both BAC and EMB (Fig. 2 and Tables S2A and B, S3A and B, and S4A and B). Largely due to this observed overlap, the same 11 PANTHER GO pathways associated with 11 putative coding DNA sequence (CDS) targets of sRNAs were identified for both life stage comparisons between the two aphid host plant treatments (BAC and EMB) (Table 2). Five of these GO pathways are associated with essential amino acid biosynthesis, specifically the arginine, chorismate, isoleucine, threonine, and valine biosynthesis pathways (Table 2). The vitamin B_6_ metabolism GO pathway was also found to be targeted by a differentially expressed sRNA (Table 2). For the comparison of ALF-BAC to FB-BAC, there are three sRNAs (*aroA_1*, *ilvI_1*, and *thrC_3*) that were significantly expressed in the bacteriocyte comparison that were not significantly expressed in the embryo comparison of ALF-EMB to FB-EMB. Similarly, in the comparison of ALF-EMB to FB-EMB, there are three sRNAs (*ilvI_2*, *thrB_2*, and *thrB_3*) that were significantly expressed in the embryo comparison that were not significantly expressed in the bacteriocyte comparison of ALF-BAC to FB-BAC (Table 2).

**TABLE 2:** Shared PANTHER GO pathways for predicted target CDSs of differentially expressed sRNAs between host plant comparisons^*a*^

Pathway associated with predicted sRNA target	Differentially expressed sRNA	Host plant treatment in which the sRNA is upregulated	
		ALF-BAC vs FB-BAC	ALF-EMB vs FB-EMB
**Arginine biosynthesis**	Antisense *carb_2*	FB	FB
**Chorismate biosynthesis**	Antisense *aroa_1*	ALF	n.s.
	Antisense *aroc*	FB	FB
*De novo* pyrimidine ribonucleotides biosynthesis	Antisense *carb_2*	FB	FB
*De novo* pyrimidine deoxyribonucleotide	Antisense *yfhc_2*	FB	FB
**Isoleucine biosynthesis**	Antisense *ilvc_2*	ALF	ALF
	Antisense *ilvd*	FB	FB
	Antisense *ilvi_1*	FB	n.s.
	Antisense *ilvi_2*	n.s.	FB
	Antisense *ilvi_5*	FB	FB
Peptidoglycan biosynthesis	Antisense *murd_1*	ALF	ALF
	Antisense *murd_3*	ALF	ALF
Salvage pyrimidine deoxyribonucleotides	Antisense *yfhc_2*	FB	FB
Salvage pyrimidine ribonucleotides	Antisense *yfhc_2*	FB	FB
**Threonine biosynthesis**	Antisense *thrb_2*	n.s.	FB
	Antisense *thrb_3*	n.s.	FB
	Antisense *thrc_3*	FB	n.s.
**Valine biosynthesis**	Antisense *ilvc_2*	ALF	ALF
	Antisense *ilvd*	FB	FB
	Antisense *ilvi_1*	FB	n.s.
	Antisense *ilvi_2*	n.s.	FB
	Antisense *ilvi_5*	FB	FB
Vitamin B_6_ metabolism	Antisense *thrc_3*	FB	n.s.

a Pathways in bold are related to essential amino acid biosynthesis. “n.s.” indicates that the specific sRNA was not differentially expressed in one of the host plant comparisons. Note that some sRNAs are present in more than one pathway.

10.1128/mBio.01733-19.5 TABLE S4 Differentially expressed intergenic sRNAs. (A) Differentially expressed intergenic sRNAs identified in the comparison of ALF-BAC to FB-BAC. (B) Differentially expressed intergenic sRNAs identified in the comparison of ALF-EMB to FB-EMB. (C) Differentially expressed intergenic sRNAs identified in the comparison of ALF-BAC to ALF-EMB. (D) Differentially expressed intergenic sRNAs identified in the comparison of FB-BAC to FB-EMB. Download Table S4, XLSX file, 0.02 MB. Copyright © 2019 Thairu and Hansen. 2019 Thairu and Hansen This content is distributed under the terms of the Creative Commons Attribution 4.0 International license.

In the comparison of ALF-BAC to FB-BAC, 7 of the 11 sRNAs associated with the PANTHER GO pathways were upregulated in FB-BAC (Table 2). The antisense sRNAs *aroA_1*, *ilvC_2*, *murD_1*, and *murD_2* were upregulated in ALF-BAC. In the comparison of ALF-EMB to FB-EMB, 8 of the 11 sRNAs predicted to target CDSs were upregulated in the FB-EMB samples, with the antisense sRNAs *ilvC_2*, *murD_1*, and *murD_3* being upregulated in the ALF-EMB samples (Table 2). Of the 14 differentially expressed sRNAs found within both comparisons, only the antisense sRNA *ilvD* was found to be conserved among the *Buchnera* lineages of A. pisum, Uroleucon ambrosiae, and Schizaphis graminum (33) (Table S2A). Eight out of 14 sRNAs are unique to the A. pisum *Buchnera* lineage and were observed previously in the strains 5A and LSR1 (33) (Table S2A). The antisense sRNAs *aroC*, *ilvI_2*, *prsA*, *thrB_2*, and *thrB_3* were identified for the first time in this study for the A. pisum *Buchnera* strain LSR1 (33) (Table S2A).

Antisense tRNA expression has been previously observed within *Buchnera* (34, 42). Within this study, more than 60% of the differentially expressed tRNAs were expressed in the antisense direction (Table 1 and Table S6A and B). All antisense tRNAs were upregulated in the FB host plant treatments for both comparisons of ALF-BAC to FB-BAC and ALF-EMB to FB-EMB, and of these antisense sRNAs, four (28%) (antisense asparagine, histidine, glutamate, and methionine tRNAs) are conserved in multiple *Buchnera* lineages (42) (Table S6A and B).

10.1128/mBio.01733-19.7 TABLE S6 Differentially expressed tRNA sRNAs. (A) Differentially expressed tRNA sRNAs identified in the comparison of ALF-BAC to FB-BAC. (B) Differentially expressed tRNA sRNAs identified in the comparison of ALF-EMB to FB-EMB. (C) Differentially expressed tRNA sRNAs identified in the comparison of ALF-BAC to ALF-EMB. (D) Differentially expressed tRNAs identified in the comparison of FB-BAC to FB-EMB. Download Table S6, XLSX file, 0.01 MB. Copyright © 2019 Thairu and Hansen. 2019 Thairu and Hansen This content is distributed under the terms of the Creative Commons Attribution 4.0 International license.

### *Buchnera* sRNAs are differentially expressed between life stages when aphids feed on either host plant.

To determine if *Buchnera* sRNA expression is different between life stages, we analyzed the following sRNA expression profiles for each host plant treatment: (i) ALF-BAC compared to ALF-EMB and (ii) FB-BAC compared to FB-EMB. For the ALF host plant treatment, one sRNA was upregulated in the ALF-BAC life stage and the remainder, 29 sRNAs, were upregulated in the ALF-EMB life stage (*q* ≤ 0.05; 1.5-fold change [Table 3]). This pattern of more sRNAs being upregulated in the EMB than in the BAC life stage was also observed in the FB host plant treatment, where 222 sRNAs were upregulated in the FB-EMB life stage and 15 were upregulated in the FB-BAC life stage (*q* ≤ 0.05; 1.5-fold change [Table 3]. Notably, there was a high level of overlap for the sRNAs that were differentially expressed between life stages for both host plant comparisons. Of the 30 sRNAs differentially expressed in the comparison of ALF-BAC to ALF-EMB, 83% were also differentially expressed, in the same direction for the comparison of FB-BAC to FB-EMB (Fig. 2 and Tables S2C and D, S3C and D, S4C and D, and S5C and D).

**TABLE 3:** Number of sRNAs upregulated for each aphid life stage comparison (*q* ≤ 0.05; 1.5-fold change)

Comparison	Type of sRNA	Upregulated in BAC	Upregulated in EMB
ALF-BAC vs ALF-EMB	Antisense sRNA	0	22
	Intergenic sRNA	1	3
	UTR sRNA	0	1
	tRNAs		
	Sense	0	0
	Antisense	0	3
	Total	1	29
FB-BAC vs FB-EMB	Antisense sRNA	12	171
	Intergenic sRNA	1	27
	UTR sRNA	1	6
	tRNAs		
	Sense	1	4
	Antisense	0	14
	Total	15	222

10.1128/mBio.01733-19.6 TABLE S5 PANTHER GO complete biological processes of the predicted CDS for differentially expressed sRNAs. (A) PANTHER GO complete biological processes of the predicted CDS for differentially expressed sRNAs in the aphid host plant comparisons that had significantly stable secondary-structure predictions. (B) PANTHER GO complete biological processes of the predicted CDS for differentially expressed sRNAs in the life stage comparisons that had significantly stable secondary-structure predictions. Download Table S5, XLSX file, 0.02 MB. Copyright © 2019 Thairu and Hansen. 2019 Thairu and Hansen This content is distributed under the terms of the Creative Commons Attribution 4.0 International license.

For the comparison of ALF-BAC to ALF-EMB, the antisense sRNAs *ilvI_1* and *ilvI_5* were predicted to target CDSs within the PANTHER GO pathways for the isoleucine and valine biosynthesis pathways. In the comparison of FB-BAC to FB-EMB, 15 antisense sRNAs, which were all upregulated in the FB-EMB samples, were predicted to target CDSs within 16 PANTHER GO pathways (Table 4). The antisense sRNAs *aroA_1*, *aroC*, *carB_1*, *ilvD*, *ilvI_1*, and *ilvI_5* were predicted to target CDSs within five PANTHER GO pathways that are associated with essential amino acids (arginine, chorismate, isoleucine, threonine, and valine) (Table 4). The antisense sRNA *serC_2* was predicted to target three GO pathways, including the vitamin B_6_ pathway. Of these 15 antisense sRNAs, 3 (*carB-1*, *ilvD*, and *pta*) are conserved across two or more *Buchnera* lineages (33) (Table S2D). The remaining 12 antisense sRNAs are specific to the A. pisum *Buchnera* lineages, with antisense the sRNAs *aroA_1*, *ilvI_1*, *ilvI_5*, and *murD_3* being conserved in both the *Buchnera* A. pisum strains 5A and LSR1 (33) (Table S2D).

**TABLE 4:** PANTHER GO pathways for predicted target CDSs of differentially expressed sRNAs between FB life stage comparisons^*a*^

Pathway associated with predicted sRNA target	Upregulated sRNA	Life stage group in which the sRNA is upregulated
Acetate utilization	Antisense *pta*	EMB
**Arginine biosynthesis**	Antisense *carb_1*	EMB
**Chorismate biosynthesis**	Antisense *aroa_1*	EMB
	Antisense *aroc*	EMB
*De novo* purine biosynthesis	Antisense *pura*	EMB
*De novo* pyrimidine ribonucleotides biosynthesis	Antisense *carb_1*	EMB
*De novo* pyrimidine deoxyribonucleotide biosynthesis	Antisense *dut_1*^*b*^	EMB
*De novo* pyrimidine deoxyribonucleotide	Antisense *yfhc_2*	FB
**Isoleucine biosynthesis**	Antisense *ilvd*	EMB
	Antisense *ilvi_1*^*b*^	EMB
	Antisense *ilvi_5*^*b*^	EMB
Peptidoglycan biosynthesis	Antisense *murd_1*	EMB
	Antisense *murd_3*	EMB
Salvage pyrimidine deoxyribonucleotides	Antisense *yfhc_2*	EMB
Salvage pyrimidine deoxyribonucleotides	Antisense *yfhc_2*	EMB
Pyridoxal-5-phosphate biosynthesis	Antisense *serc_2*	EMB
Serine glycine biosynthesis	Antisense *serc_2*	EMB
**Threonine biosynthesis**	Antisense *thrb_3*	EMB
**Valine biosynthesis**	Antisense *ilvd*	EMB
	Antisense *ilvi_1*^*b*^	EMB
	Antisense *ilvi_5*^*b*^	EMB
Vitamin B6 metabolism	Antisense *serc_2*	EMB

a Pathways in bold are related to essential amino acid biosynthesis. Note that some sRNAs are present in more than one pathway.

b Antisense sRNAs *dut_1*, *ilvI_1*, and *ilvI_5* were also detected in the comparison of ALF-BAC to ALF-EMB and were upregulated in the EMB life stage group. None of the other sRNAs targeting CDS in the PANTHER GO pathways listed were differentially expressed in the comparison of ALF-BAC to ALF-EMB, and therefore a column was not included for this comparison.

Antisense tRNAs made up the majority of the differentially expressed tRNAs identified between life stage comparisons for both aphid host plant treatments. Within the comparison of ALF-BAC to ALF-EMB, three differentially expressed antisense tRNAs were upregulated in the ALF-EMB samples (Table S6C). One of these antisense tRNAs (glutamate) was also identified in the comparisons of ALF-BAC to FB-BAC and ALF-EMB to FB-EMB and is conserved among multiple *Buchnera* lineages (42). In the comparison of FB-BAC to FB-EMB, 14 antisense tRNAs were differentially expressed and upregulated in the FB-EMB samples (Table S6D). Four of these antisense tRNAs (asparagine, histidine, glutamate, and methionine tRNAs), which were also identified in the comparisons of ALF-BAC to FB-BAC and ALF-EMB to FB-EB, are conserved among *Buchnera* lineages (42) (Table S6C and D).

## DISCUSSION

This study demonstrated for the first time that *Buchnera* sRNAs are differentially expressed between host plant species and potentially target genes within essential amino acid biosynthesis pathways. The results from this study also suggest that even though sRNAs are differentially expressed between *Buchnera* life stages (34; this study), sRNA expression profiles are heavily influenced by what host plant species the aphid feeds upon. This study also found that 21% and 26% of *Buchnera* sRNAs that are differentially expressed between aphid host plant species and between life stages, respectively, are conserved in two or more *Buchnera* taxa. It is unclear at this time if *Buchnera* displays differential protein expression when aphids feed on different host plants, and if putative differential protein expression is linked to the candidate CDS targets for sRNAs identified in this study. It will be of interest for future studies to address whether these sRNA candidates are important in facilitating posttranscriptional regulation in *Buchnera* when aphids feed on different host plant diets.

**FIG 3:**
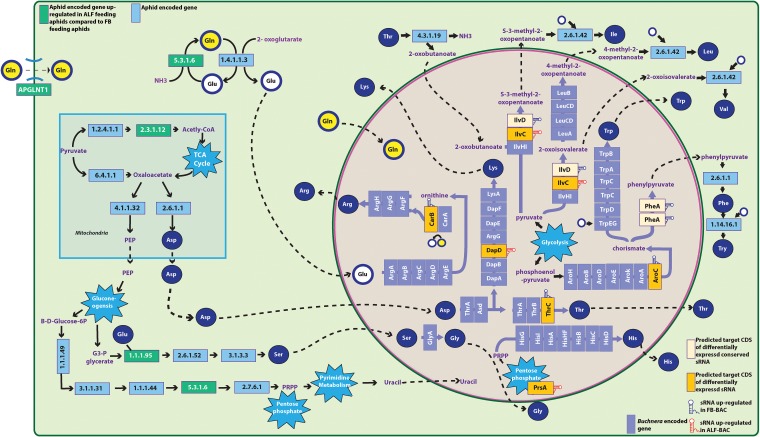
Integration of amino acid biosynthetic pathways of the aphid and *Buchnera* within the aphid bacteriocyte. Yellow boxes indicate the predicted CDS targets of differentially expressed *Buchnera* sRNAs identified in the comparison of ALF-BAC to FB-BAC. Green and purple areas represent the cytosol of the bacteriocyte and of the *Buchnera* cell, respectively. Green and purple lines represent aphid and *Buchnera* cell membranes, respectively. Amino acids are represented by blue disks or by yellow (glutamine) or white (glutamate) disks with blue outlines. Aphid gene expression data adapted from the work of Kim et al. (21). *Buchnera* sRNA expression data are from this study.

*Buchnera* sRNA expression is more heterogenous among biological replicates in both life stages when A. pisum feeds on its specialized host plant alfalfa than when it feeds on its universal host plant, fava bean (Fig. 1). It is uncertain if this heterogenous response is driven by one of the aphid sublines or is a variable response to its specialized host plant, alfalfa. Despite this diffuse response in sRNA expression profiles, there is a significant difference in sRNA expression observed between host plant treatments for both life stages, and no batch effects were observed. We hypothesize that the potential variation of metabolites (which include amino acids and plant defense compounds) in alfalfa may contribute to the heterogeneity observed in *Buchnera* sRNA expression profiles when aphids feed on alfalfa. The two A. pisum host plants used in this study are known to vary significantly in essential amino acid profiles (35) and metabolite profiles, including host plant defense compounds (38, 39). Aphid stylectomy experiments have also shown that fava bean and alfalfa have different concentrations of arginine, isoleucine, leucine, lysine, phenylalanine, tryptophan, tyrosine, valine, and histidine, with alfalfa generally having lower concentrations of essential amino acids than fava bean (35). Regardless of *Buchnera* life stage, sRNAs predicted to target genes within the arginine, chorismate (phenylalanine, tryptophan, and tyrosine), isoleucine, threonine, and valine biosynthesis pathways are differentially expressed between aphid host plant treatments, which corresponds to the difference in host plant amino acid content (Table 2, Fig. 3, and Fig. S1). Previous studies have shown that A. pisum aphids that feed on alfalfa display lower mass than that of aphids that feed on fava bean, which is suggestive that they obtain fewer nutrients and/or expend more energy when they feed on alfalfa (21, 43, 44). This variation in aphid mass could also be due to the variation in metabolites that act as feeding deterrents in alfalfa. When comparing the metabolite profiles among the A. pisum host plants alfalfa, fava bean, pea, and red clover, Sanchez-Arcos et al. (38) found that alfalfa plants contain the highest number of unique metabolites, especially compared to that of fava bean plants. Notably, Sanchez-Arcos et al. (38) found that saponins, a known A. pisum feeding deterrent (43, 44), were specific to alfalfa and the most abundant plant chemical compound within this host plant. An alternative hypothesis to explain this host plant-specific pattern is that *Buchnera* sRNAs may respond sporadically, in a nondirected manner, if *Buchnera* is stressed when the aphid feeds on a metabolically complex diet such as alfalfa (43, 44). Of the 322 differentially expressed sRNAs identified in this study, 18% are predicted to target genes that are associated with stress responses in Escherichia coli (45). Future work is needed to determine if the observed aphid host plant-specific response of *Buchnera* sRNAs results in the differential expression of predicted *Buchnera* protein targets, which potentially can impact this symbiosis in either an adaptive nutritional manner or a nonadaptive random stress response.

10.1128/mBio.01733-19.1 FIG S1 Predicted CDS targets of the differentially expressed sRNAs found in the comparison of ALF-EMB to FB-EMB. Orange boxes indicate the predicted CDS targets of differentially expressed *Buchnera* sRNAs identified. Green and pink areas represent cytosol of the bacteriocyte and of a *Buchnera* cell, respectively. Green and pink lines represent aphid and *Buchnera* membranes, respectively. Amino acids are represented by blue disks or by yellow (glutamine) or white (glutamate) disks with blue outlines. Download FIG S1, PDF file, 0.4 MB. Copyright © 2019 Thairu and Hansen. 2019 Thairu and Hansen This content is distributed under the terms of the Creative Commons Attribution 4.0 International license.

In the life stage comparisons of ALF-BAC to ALF-EMB and FB-BAC to FB-EMB, there was notable overlap in the differentially expressed antisense sRNAs identified for both host plant treatments (Fig. 2 and Table S2C and D). The differentially expressed antisense sRNAs found in both life stage comparisons may potentially be crucial in the regulation of important life stage-specific CDSs regardless of host plant diet. Of these 22 overlapping antisense sRNAs, 6 (*ansA_1*, *alaS_2*, *leuS*, *ilvI_1*, *ilvI_5*, and *truB_1*) had significant thermodynamically stable secondary-structure prediction. The presence of thermodynamically stable secondary structures in part suggests that these sRNAs may potentially have a functional role (46). Other than the antisense sRNAs *ilvI_1* and *ilvI_5*, which were predicted to target CDS within the isoleucine and valine biosynthesis PANTHER GO pathways, the remaining four antisense sRNAs were predicted to target genes in eight PANTHER GO biological processes, which include mRNA pseudouridine synthesis and pseudouridine synthesis (Table S5). *Buchnera* is unable to produce its own uracil, a precursor for pseudouridine synthesis, and therefore, it depends on the host for uracil biosynthesis (13) (Fig. 3). Pseudouridine is a widespread and functionally important posttranscriptional modification of RNAs within eukaryotes (47). Within E. coli, not only can pseudouridine modification of mRNAs influence translation, reducing protein expression (48), but also the presence of pseudouridine in a stop codon can result in readthrough (49). It is therefore possible that pseudouridine modification is important in *Buchnera* to facilitate different translation rates between life stages. In the comparisons of ALF-BAC to ALF-EMB and FB-BAC to FB-EMB, there was also a trend in which more sRNAs were upregulated in the EMB life stage than in the BAC life stage (Table 3). A similar trend was also observed in previous experiments comparing *Buchnera* sRNA expression with the EMB and BAC aphid life stages (34). One key difference between these two aphid life stages is that within the EMB life stage, *Buchnera* transitions from an extracellular proliferating state within the aphid embryos to an intracellular nonproliferating one in the aphid bacteriocytes (41). The difference in sRNA expression between the two life stages suggests that dynamic gene regulation is required during the extracellular transitionary period of the aphid embryo in contrast to the intracellular state within the homeostatic environment of the aphid bacteriocyte.

Insects, like many other animals, cannot synthesize B vitamins *de novo*, and so they obtain these necessary micronutrients from their diet or their association with various microorganisms (50). Within aphids, *Buchnera* possesses several genes for the biotin (B_7_), folic acid (B_9_), pantothenate (B_5_), pyridoxine (B_6_) riboflavin (B_2_), and thiamine (B_1_) biosynthesis pathways (13, 51). Vitamin B_6_ is an important bacterial and eukaryotic cofactor, especially in amino acid metabolism (52, 53), and neither *Buchnera* nor A. pisum has all the genes needed for its biosynthesis. Previous work looking at aphid gene expression has shown that within bacteriocytes of A. pisum that have fed on alfalfa, the vitamin B_6_ pathway was significantly enriched (21). In this study, it was found that in the comparison of ALF-BAC to FB-BAC, the antisense sRNA predicted to target *thrC*, one of the two *Buchnera* genes vital for vitamin B_6_ biosynthesis, was upregulated in the ALF-BAC samples (Table S2A). In addition, a second antisense sRNA, which was upregulated in the FB-EMB samples compared to the FB-BAC samples, was predicted to target *serC*, the other *Buchnera* gene vital for vitamin B_6_ biosynthesis. Further studies that directly manipulate vitamin B nutrition, in conjunction with proteomics, will help illuminate whether the antisense sRNAs *thrC* and *serC* differently regulate their predicted CDSs.

One of the most important tasks in the field of bacterial sRNA research is determining if a particular putative sRNA has a functional role. Working in nonmodel, nonculturable systems, such as *Buchnera*, increases this challenge because many of the techniques used to validate and determine functionality are not easily implemented or possible (30). Nevertheless, the results from this study are consistent with previous *Buchnera* sRNA experiments, which provide support for the potential function of sRNA candidates. For example, Thairu et al. (34) observed *Buchnera* sRNA expression between bacteriocytes and embryos of aphids that fed on fava bean. Thairu and colleagues (34) identified 90 differentially expressed sRNAs, 27 of which were also identified in the comparison of FB-BAC to FB-EMB conducted in this study (34) (Tables S2D, S3D, S4D, and S6D). Both studies also identified sRNAs predicted to target CDSs within similar PANTHER GO pathways, specifically, the acetate utilization, arginine biosynthesis, *de novo* purine biosynthesis, *de novo* pyrimidine ribonucleotide biosynthesis, isoleucine biosynthesis, peptidoglycan biosynthesis, and valine biosynthesis pathways. Though the two studies used the same aphid lines, methodological differences, such as different insect rearing locations and differences in sequencing platforms, library preparation, and centers, could account for some the differences observed.

In the report by Thairu et al. (34), the authors provided evidence for the functionality of the antisense sRNA *carB* (*carB_1* in this study) by heterologously expressing the sRNA in E. coli. This study not only detected this sRNA but also found that it had the same expression pattern as described by Thairu et al. (34) between different *Buchnera* life stages. There was also an overlap between this study and the proteomic study conducted by Hansen and Degnan (33), who characterized the differentially expressed *Buchnera* proteins between *Buchnera* life stages when aphids feed on fava bean. Specifically, 27 of the 54 differentially expressed proteins identified by Hansen and Degnan (33) were either directly or indirectly (i.e., within an operon) associated with a differentially expressed sRNAs identified in this study (Table S7). This study also found 72 (53 antisense sRNAs, 15 UTR sRNAs, and 4 intergenic sRNAs) differentially expressed conserved sRNAs, which were also characterized by Hansen and Degnan (33) (Tables S2, S3, and S4). The conservation of sRNAs across four *Buchnera* lineages (*Buchnera* A. pisum, Acyrthosiphon kondoi, Uroleucon ambrosiae, and Schizaphis graminum) that diverged over 65 million years ago strongly suggests that some sRNAs may be important in *Buchnera*’s gene regulation. Recent genomic studies further support the hypothesis that sRNA regulation is likely conserved across *Buchnera* strains. Specifically, Chong et al. (54) recently compared the genomes of 39 *Buchnera* strains and found that across these taxa, 29 genes are under strong positive selection within the aphid subfamily Aphidinae. Interestingly, of these 29 genes, there is evidence that 24 are predicted targets of conserved antisense or UTR sRNAs (33). Twenty of the 29 *Buchnera* genes under positive selection (54) were predicted to be either direct or indirect targets of one or more differentially expressed antisense sRNAs identified in this study. Four of these genes (*asps*, *mtlA*, *rnr*, and *serC*) were also found to be differentially expressed as proteins in *Buchnera* embryos and maternal bacteriocytes, which corresponds to differential expression patterns of antisense sRNA identified in this and other studies (33, 34).

10.1128/mBio.01733-19.8 TABLE S7 Operons that have both sRNA upregulation and protein upregulation in the *Buchnera* life stage comparisons in the FB aphid host plant treatment. Download Table S7, XLSX file, 0.01 MB. Copyright © 2019 Thairu and Hansen. 2019 Thairu and Hansen This content is distributed under the terms of the Creative Commons Attribution 4.0 International license.

In recent years, increasing numbers of studies have begun to find sRNAs being expressed in highly reduced genomes, including organelles (30, 55). Though the roles of sRNAs within these reduced genomes are still poorly understood, within mitochondria and chloroplasts they have been described to respond to stress (56–59) and host development and/or tissue type (60–63). Based on the trends observed in other reduced genomes and the patterns that we observed within the *Buchnera* system, we hypothesize that sRNAs can be maintained and evolve in reduced genomes potentially to help compensate for the loss of regulatory proteins. Host-restricted bacterial symbionts with highly eroded genomes, such as *Buchnera*, face the challenge of having to compensate for the loss of key genomic material, and until recently, many of the compensatory methods that have been described have been only been host meditated. Moving forward, based on our data and other studies, especially since positive selection can occur in these reduced genomes (54), we predict that more examples of symbiont mediated control will emerge.

## MATERIALS AND METHODS

### Small-RNA sample preparation and sequencing.

Three sublines of A. pisum (LSR1) that were established by Kim et al. (21) were allowed to independently develop and feed for >100 generations on either V. faba (fava bean [FB]) or M. sativa (alfalfa [ALF]) (*n* = 3 biological replicates per host plant species treatment). These six sublines were reared in a growth chamber at 20°C under a 16-h light/8-h dark regime.

For each subline treatment, two life stage samples were cocollected from the same 4th-instar aphid nymph individual via dissection, similar to the method of Thairu et al. (34). These two samples represent two different *Buchnera* life stages: (i) aphid ovarioles (EMB) and (ii) maternal bacteriocytes (BAC). For each sample, approximately 200 aphids from each subline were dissected for each life stage and pooled. In total, 12 samples were collected: three bacteriocyte biological replicate samples from aphids feeding on alfalfa (ALF-BAC), three bacteriocyte biological replicate samples from aphids feeding on fava bean (FB-BAC), three embryo biological replicate samples from aphids feeding on alfalfa (ALF-EMB), and three embryo biological replicate samples from aphids feeding on fava bean (FB-EMB). All tissues were immediately stored in RNAprotect bacterial reagent (Qiagen, Germantown, MD) and stored at −80°C.

For each sample (*n* = 12), RNA was extracted using an miRNAeasy kit (Qiagen, Germantown, MD). Library preparation and sequencing were then performed on the small-RNA-enriched fraction (≤250 nucleotides [nt]) using the Illumina (San Diego, CA) mRNA directional sequencing protocol by the University of California, San Diego, Institute for Genomic Medicine Genomics Center (UCSD IGM Genomics Center). Each library was then sequenced as 75-nt single-end reads on an Illumina Hi-seq 4000 at the UCSD IGM Genomics Center.

### Identification and categorization of *Buchnera* sRNAs.

Reads for each sample were independently quality screened using Trimmomatic v.0.33 (64). Adapters were then removed using Cutadapt v.2.1 (65). To remove aphid reads, sequences were aligned to the aphid genome (GCA_000142985.2) using Bowtie2 v.2.2.9 (66). Once aphid reads were removed, Bowtie2 was used to map the remaining reads to the *Buchnera* genome (GCA_000174075.1). For all Bowtie2 runs the default settings were used (66). Rockhopper v.2.0.3 (67) was then used to identify putative *Buchnera* sRNAs in each sample. The Rockhopper parameters optimized for identifying *Buchnera* sRNAs by Hansen and Degnan (33) were used to identify sRNAs in this study. Reads were normalized by the upper-quartile method in Rockhopper. sRNA boundaries were manually determined by inspecting directional coverage curves in Artemis v.16 (68). sRNAs were then binned into three different categories, similar to those of Hansen and Degnan (33) and Thairu et al. (34): (i) sRNAs expressed antisense to the gene (antisense sRNAs), (ii) sRNAs expressed within the untranslated regions of genes (UTR sRNAs), and (iii) sRNAs identified within the intergenic spacer regions (intergenic sRNAs). As in the works of Hansen and Degnan (33) and Thairu et al. (34), antisense and UTR sRNAs are named after their predicted target coding sequence (CDS) based on direct base-pairing interactions. For example, the antisense sRNA *aroC*, which is expressed antisense to the *aroC* coding sequence, is predicted to target the CDS *aroC*. If multiple sRNAs are predicted to target different regions of the same CDS, then a number follows (e.g., antisense sRNAs *ilvI_1* and *ilvI_2*). For sRNAs expressed within the intergenic spacer region, the sRNA name contains both the upstream and downstream CDS names (e.g., intergenic sRNA *argH-yibN*).

Though target binding and functionality of the putative *cis-*acting *Buchnera* sRNAs identified in this study have not been determined, we predict that they would function similarly to *cis*-acting antisense sRNAs in free-living bacterial systems (see reviews in references [Bibr B69][Bibr B70][Bibr B71]). Previous work with *Buchnera* has validated the functionality of an expressed antisense sRNA *in vitro* using heterologous expression (34). Further, in the work of Hansen and Degnan (33), 52 protein targets showed evidence of posttranscriptional regulation, which may be mediated by these types of *cis*-RNA interactions.

Using the read counts for each of the identified sRNAs from Rockhopper, a principal-component analysis (PCA) was conducted in R v.3.5.2 (72) using the package DEbrowser v.1.10.6 (73) to compare how similar *Buchnera* sRNA expression profiles were across all treatments. The reads were filtered using the default DEbrowser settings and were normalized by the upper-quartile method, the same method used by Rockhopper (67). The following four groups were compared: ALF-BAC, ALF-EMB, FB-BAC, and FB-EMB. Using R, a permutational multivariate analysis of variance (PERMANOVA) was used to determine if sRNA profiles were significantly different between treatments (74).

Rockhopper was also used to determine if sRNAs were differentially expressed between host plant treatments and life stage categories. sRNA expression comparisons include (i) bacteriocytes from aphids feeding on alfalfa (ALF-BAC) compared to bacteriocytes from aphids feeding on fava (FB-BAC), (ii) embryos from aphids feeding on alfalfa (ALF-EMB) compared to embryos from aphids feeding on fava (FB-EMB), (iii) bacteriocytes compared to embryos from aphids feeding on alfalfa (ALF-BAC and ALF-EMB, respectively), and (iv) bacteriocytes compared to embryos from aphids feeding on fava (FB-BAC and FB-EMB, respectively). Significance criteria of a ≥1.5-fold change between samples and a *q *of <0.05 were used to determine if an sRNA was differentially expressed. The putative secondary structures were predicted for all differentially expressed sRNAs using RNAalifold v.2.1 (75, 76), following the same methods as Hansen and Degnan (33).

Using PANTHER GO functional gene list analysis (77), the GO pathways and GO biological processes were identified for the predicted CDS targets of sRNAs that displayed both significant differential expression and predicted thermodynamic stability. Only putative *cis-*acting sRNAs (e.g., antisense and UTR sRNAs, which have hypothetical direct base-pairing interactions with their CDS target) were included in this analysis, because it is uncertain whether putative intergenic sRNAs target one or both neighboring genes or act in *trans* and target other genes located distantly in the genome.

### Accession number(s).

The sequence data for RNA-seq reads from all samples were submitted to the NCBI database under BioProject accession number PRJNA549470.
