# Supplementary material for: Changes in Aphid Host Plant Diet Influence the Small-RNA Expression Profiles of Its Obligate Nutritional Symbiont, Buchnera
Source: mBio. 2019 Nov 19;10(6):e01733-19. doi: 10.1128/mBio.01733-19 (PMC6867890; doi:10.1128/mBio.01733-19)
Supplement: TEXT S1 [file mBio.01733-19-s0001.docx]

**Supplementary Results**

*Determination of batch effects within samples:*

Combat and Harmen batch effect correction tests were run using DeBrowser (Kucukural et al., 2019). All data was normalized via upper-quartile normalization. The results show that before the correction methods are utilized samples group by aphid tissue-type (i.e. embryos [EMB] and bacteriocytes [BAC]), not by any type of experimental batch such as each co-collected tissues sample e.g. ALF-BAC1 and ALF-EMB1(Supplemental Figure 1.1). These results also show that there is no library effect, since both bacteriocyte and embryo samples from a single host-plant treatment were prepared and run on the same Illumina lane. Overall, this indicates that batch effects are not strongly influencing the data set, and the differences observed are due to biological differences.

*Aphid host-plant diet significantly affects* Buchnera *sRNA expression for genes involved in essential amino acid biosynthesis:*

Within the BAC life-stage 69 antisense sRNAs were up-regulated in ALF host-plant treatment and 17 were up-regulated in the FB host-plant treatment (Table 1). Of these differentially expressed antisense sRNAs 53% were found to have significantly greater predicted thermodynamic stability and sequence covariation than randomized alignments in RNAalifold, suggesting putative regulatory and/or structural function (one-tailed t-test, d.f.=99, P<0.05; Supplemental Table 2A). In the ALF-EMB *vs.* FB-EMB comparison, 63 antisense sRNAs were up-regulated in the ALF host-plant treatment and 56 antisense sRNAs up-regulated in the FB host-plant treatment with 45% of them showing significant thermodynamic stability (one-tailed t-test, d.f.=99, P<0.05; Table 1; Supplemental Table 2B). Within these comparisons of ALF-BAC vs. FB-BAC and ALF-EMB vs. FB-EMB, the majority (70% and 53% respectively) of these differentially expressed antisense sRNAs have also been identified in previous studies (Hansen & Degnan 2014; Thairu et al. 2018; Supplemental Table 2A-B). In the BAC life-stage comparison (ALF-BAC *vs.* FB-BAC), 22 of the previously identified antisense sRNAs were found in two or more *Buchnera* lineages; the remaining 39 sRNAs were identified in two *A. pisum, Buchnera* strains, 5A and LSR1 (Supplemental Table 2A). For the EMB life-stage comparison (ALF-EMB *vs.*FB-EMB), 48 of the previously identified antisense sRNAs were found in the two *A. pisum, Buchnera* strains 5A and LSR1, the remaining 16 antisense sRNAs were found in two or more *Buchnera* lineages (Supplemental Table 2B).

UTR sRNAs can be found within either the 3’ or 5’UTR of a gene. For the ALF-BAC *vs.* FB-BAC comparison, the 11 sRNAs that were differentially expressed in the UTR regions were all up regulated in the ALF-BAC samples (Table 1; Supplemental Table 3A). Eight of these sRNAs were up-regulated in the 3’ UTR region and three up-regulated in the 5’ UTR region (Supplemental Table 3A). The sRNAs expressed in the 5’ UTR region of *fpr* and *mutL,* were found to be expressed in the antisense orientation of the coding sequences *fpr* and *mutL*. (Supplemental Table 3A). Sixty-three percent of the UTR sRNAs identified had a significantly greater predicted thermodynamic stability and sequence covariation than randomized alignments (one-tailed t-test, d.f.=99, P<0.05; Supplemental Table 3A). For the ALF-EMB *vs.* FB-EMB comparison, of the seven UTR sRNAs that were up-regulated in ALF-EMB samples, six mapped to the 3’ region (Table 1; Supplemental Table 3B). The one 5’ UTR sRNA that was up-regulated in the ALF-EMB samples mapped to the *cyaY* gene. The two UTR sRNAs that were up-regulated in the FB-EMB samples, both mapped to the 5’ regions the *amiB* and *htpX* genes (Table 1; Supplemental Table 3B). Sixty-six percent of the differentially expressed UTR sRNAs identified were predicted to have significant thermodynamic stability (one-tailed t-test, d.f.=99, P<0.05; Supplemental Table 3B). Of the differentially expressed UTR sRNAs, 72% and 88%, (ALF-BAC *vs.* FB-BAC and ALF-EMB *vs.* FB-EMB respectively) were also identified in previous studies and predicated to be conserved in two or more *Buchnera* lineages (Hansen & Degnan 2014, Supplemental Table 3A-B).

Regarding intergenic sRNAs, 19 were up-regulated in the ALF-BAC samples and two up-regulated in the FB-BAC samples for the ALF-BAC *vs.* FB-BAC comparison (Table 1). The majority (18/21) of these differentially expressed intergenic sRNAs were also predicted to have significant thermodynamic stability (one-tailed t-test, d.f.=99, P<0.05; Supplemental Table 4A). For the ALF-EMB *vs.* FB-EMB comparison, 29 intergenic sRNAs were differentially expressed. Of these, 22 were up-regulated in the ALF-EMB samples and seven were up-regulated in the FB-EMB samples. Within ALF-EMB *vs.* FB-EMB comparison, 62% of the differentially expressed intergenic sRNAs show significant thermodynamic stability. (one-tailed t-test, d.f.=99, P<0.05; Supplemental Table 4B). Unlike the antisense sRNAs, the vast majority (46/50) of these intergenic sRNAs are novel to this study. Of the four sRNAs that were previously identified, one (*nufA-ssb*), was identified as up-regulated in the ALF-EMB sample in the ALF-EMB *vs.* FB-EMB comparison. The remaining three conserved intergenic sRNA (*cspC- yoaE,* *rplM-pheM*, and *ycfM-ompF*) were up-regulated in the ALF-BAC samples in the ALF-BAC *vs.* FB-BAC comparison. All three of these intergenic sRNAs have evidence of conservation within the two *A. pisum, Buchnera* strains 5A and LSR1(Supplemental Table 4A-B).

*Buchnera sRNAs are differentially expressed between life-stages when aphids feed on either host-plant:*

Of the 30 differentially expressed sRNAs identified between the ALF-BAC and ALF-EMB samples, all but one intergenic sRNA was upregulated in the ALF-EMB samples (Table 3). Specifically, 22 differentially expressed antisense sRNAs were identified, with six showing significant predicted thermostability (one-tailed t-test, d.f.=99, P<0.05; Supplemental Table 2C). Only one UTR sRNA was differentially expressed between the *Buchnera* life-stages in the ALF-BAC *vs.* ALF-EMB comparison (Table 3). This UTR sRNA was predicted to map to the 5’ region of *hscB* and was not thermodynamically stable (one-tailed t-test, d.f.=99, P<0.05; Supplemental Table 3C). Of four differentially expressed intergenic sRNAs, one was previously identified by Hansen & Degnan (2014) and was predicted between the gene *rplN* and *rps* (Supplemental Table 4C). All four of these differentially expressed intergenic sRNAs were also predicted to be thermodynamically stable and up-regulated in the ALF-EMB samples (one-tailed t-test, d.f.=99, P<0.05; Supplemental Table 4C).

Within the FB host-plant treatment, antisense sRNAs made up the vast majority (77%) of the differentially expressed sRNA between life-stages (Table 3). Of these 183 antisense sRNAs, 12 were up-regulated in the FB-BAC samples and 171 were up-regulated in the FB-EMB samples (Table 3). Thirty-nine percent of these differentially expressed sRNAs were found to have significant thermostability (one-tailed t-test, d.f.=99, P<0.05; Supplemental Table 2D). Of these differentially expressed antisense sRNAs, 73 have been previously identified by Hansen & Degnan (2014), (Supplemental Table 2D). Of these 71 antisense sRNAs, 43% were predicted to be conserved in two or more *Buchnera* lineages (Hansen & Degnan, 2014). Sixteen antisense sRNAs were found both in this study and Thairu et al. (2018) (Supplemental Table 2D)*.*

Seven differentially expressed UTR sRNAs were also identified within the FB-BAC *vs.* FB-EMB comparison. Of these seven UTR sRNAs, six were up-regulated in the FB-EMB samples and 57% of them were predicted to have significant thermostability (one-tailed t-test, d.f.=99, P<0.05; Supplemental Table 3D). Two UTR sRNAs were predicted to fall within the 3’ UTR of *flgK* and *fba,* and the remaining five in the 5’ UTR*.* All but the 3’ *fba* UTR sRNAs were previously identified in Hansen & Degnan (2014). Of the previously identified UTR sRNAs all but the 5’ UTR sRNA *hscB,* showed conservation across two or more *Buchnera,* lineages (Supplemental Table 3D). None of the UTR sRNAs identified in the study were found in to be differentially expressed by Thairu et al. (2018).

In the FB-BAC *vs.* FB-EMB comparison, 27 differentially expressed intergenic sRNAs were found; one of which was up-regulated in the FB-BAC treatment (Table 3). The majority (75%) of these intergenic sRNAs were predicted to have significant thermostability (one-tailed t-test, d.f.=99, P<0.05; Supplemental Table 4D). Of these 27 intergenic regions, only one (*cspC-yoaE*), overlapped directly with a previously identified intergenic sRNA (Supplemental Table 4D). None of the intergenic sRNAs identified in the study were found in Thairu et al. (2018).

*sRNAs expression patterns are congruent with protein expression patterns between bacteriocytes and embryos of aphids feeding on fava bean:*

Hansen & Degnan (2014), characterized the *Buchnera* protein expression profiles in embryos (EMB) and bacteriocytes (BAC) of aphids feeding on fava bean (FB). We therefore compared the differentially expressed sRNAs identified for the FB-BAC *vs.* FB-EMB comparisons in this study to the protein expression profiles characterized by Hansen & Degnan (2014). sRNAs can both regulate at both the gene and operon level, as such we included the operons when identifying which proteins may be regulated by differentially expressed sRNAs.

Overall, out of 54 differentially expressed proteins identified by Hansen & Degnan (2104), 27 were associated with a differentially expressed sRNA either directly or indirectly within an operon (Supplemental Table 7). Three of these proteins were predicted to be regulated by UTR sRNAs, and 24 were predicted to be regulated by antisense sRNAs. Seventeen operons were predicted to have significantly up-regulated sRNAs and proteins in the same life-stage, suggesting that these sRNAs may be involved in the activation/stabilization of these proteins. Conversely, seven operons were predicted to have sRNAs significantly up-regulated in the opposite life-stage to their predicted protein target which is suggestive that these sRNAs may be involved in the repression of their protein targets (Supplemental Table 7)*.* Specifically, four operons contained proteins that were up-regulated in the EMB life-stage while the predicted associated sRNAs were up-regulated in the BAC life-stage. Similarly, three operons were predicted to have the inverse pattern of proteins being up-regulated in the BAC-life stage and their predicted associated sRNAs up-regulated in the EMB life-stage.

**Supplemental Figure 1.1:**

A. Harmen batch correction analysis results. All samples before (A) and after (B) combat correction. Panels (C) and (D) represent bacteriocyte (BAC) and embryo (EMB) tissue samples from aphids feeding on fava bean (FB). Panels (E) and (F) resents the tissue samples from aphids feeding on alfalfa (ALF).

B. Combat batch correction analysis results. All samples before (G) and after (H) combat correction. Panels (I) and (J) represent bacteriocyte (BAC) and embryo (EMB) tissue samples from aphids feeding on fava bean (FB). Panels (K) and (L) resents the tissue samples from aphids feeding on alfalfa (ALF).

**References:**

Hansen AK, Degnan PH. 2014. Widespread expression of conserved small RNAs in small symbiont genomes. *ISME J.* 8:2490–2502.

Kucukural A, Yukselen O, Ozata DM, Moore MJ, Garber M. 2019. DEBrowser: interactive differential expression analysis and visualization tool for count data. *BMC Genomics* 20:6.

Thairu MW, Cheng S, Hansen AK. 2018. A sRNA in a reduced mutualistic symbiont genome regulates its own gene expression. *Mol. Ecol.* 27, 1766–1776.
