# Supplementary figures and images for: Changes in Aphid Host Plant Diet Influence the Small-RNA Expression Profiles of Its Obligate Nutritional Symbiont, Buchnera
Source: mBio. 2019 Nov 19;10(6):e01733-19. doi: 10.1128/mBio.01733-19 (PMC6867890; doi:10.1128/mBio.01733-19)

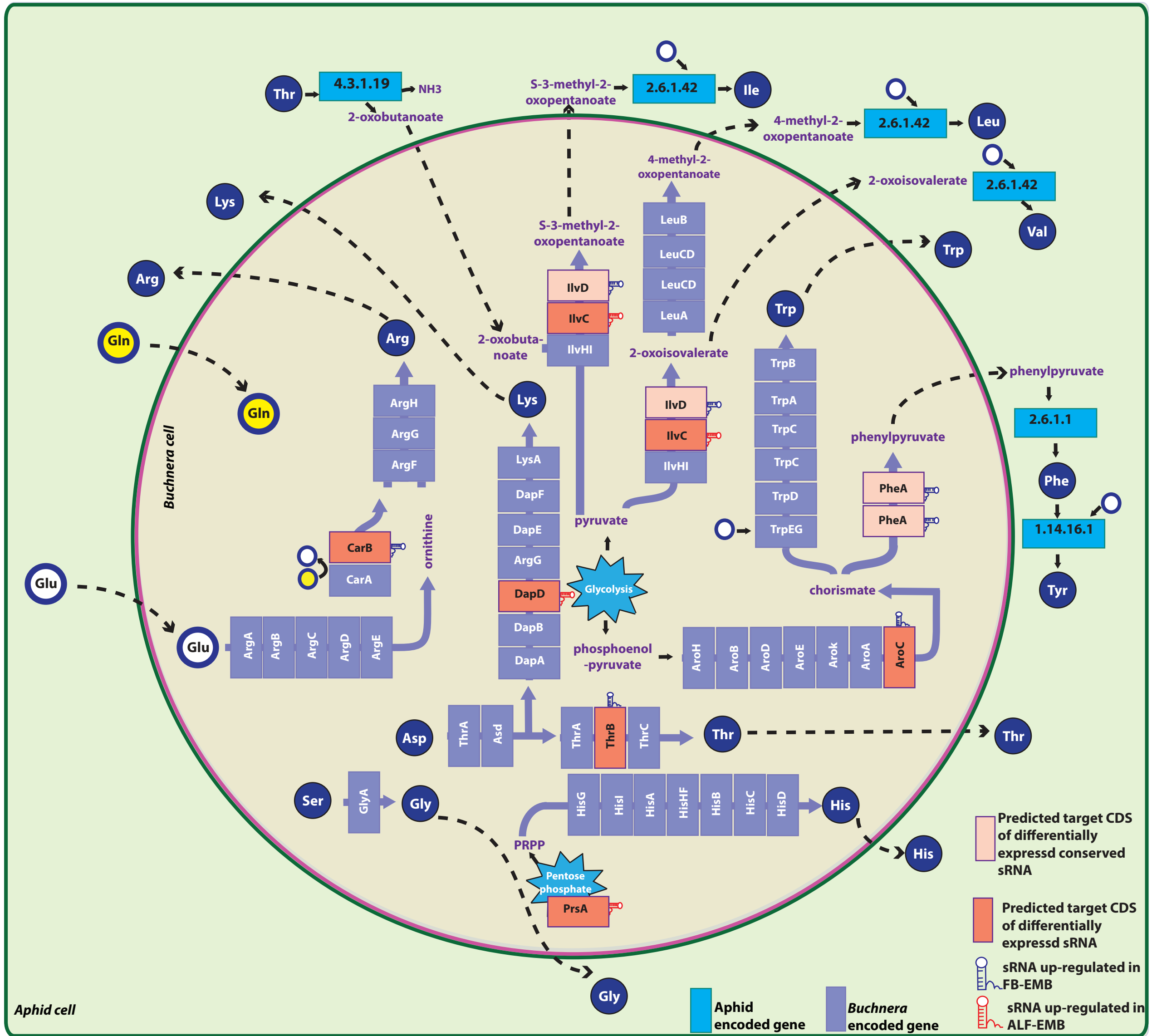

Supplement: FIG S1 [file mBio.01733-19-sf001.pdf]
